# COVID-19-Induced Colitis: A Novel Relationship During Troubling Times

**DOI:** 10.7759/cureus.15870

**Published:** 2021-06-23

**Authors:** Peter Stawinski, Karolina N Dziadkowiec, Akiva Marcus

**Affiliations:** 1 Internal Medicine, University of Miami JFK Medical Center, Atlantis, USA; 2 Gastroenterology, University of Miami, JFK Regional Campus, Atlantis, USA

**Keywords:** covid19, infectious colitis, coronavirus disease 2019 (covid-19), gastroenterology, adult gastroenterology, gastroenterology and endoscopy

## Abstract

Severe acute respiratory syndrome coronavirus 2 (SARS-CoV-2) and the resulting disease called coronavirus disease 2019 (COVID-19) has initiated a global health crisis declared by the World Health Organization (WHO). As the nature of this novel virus unfolds, there have been a variety of extra-pulmonary clinical presentations of COVID-19 affecting the gastrointestinal tract. We present a novel relationship between this disease and its clinical manifestation as COVID-19-induced colitis. Providing insight into this association will invariably facilitate earlier recognition and resultant treatment of COVID-19 along with reducing unintended exposure to healthcare workers.

## Introduction

In December 2019, a novel coronavirus was identified known as acute respiratory syndrome coronavirus 2 (SARS-CoV-2) and the resulting disease called coronavirus disease 2019 (COVID-19) [1]. In March 2020, the World Health Organization (WHO) declared this coronavirus outbreak as a global pandemic and worldwide emergency. Symptoms of COVID-19 most commonly include fever (88%), shortness of breath and cough (68%); transmission of SARS-CoV-2 is thought to occur from human to human contact via respiratory secretions [2]. As this disease course unfolds, there have been a variety of clinical presentations with extra-pulmonary manifestations of COVID-19, including a multitude of gastrointestinal symptoms, most commonly, vomiting (5%) and diarrhea (3.7%) [2]. A cross-sectional multicenter study of three heavily affected hospitals during the initial outbreak in Hubei province reported 18.6% of patients presented to the hospital with gastrointestinal-specific symptoms including anorexia, diarrhea, vomiting and abdominal pain; in fact a small number of patients presented with gastrointestinal symptoms alone [3]. Although the majority of patients with COVID-19 primarily present to the hospital with respiratory symptoms, it is particularly important that physicians, including gastroenterologists, become familiar with these rare extra-pulmonary manifestations in an effort to reduce the delay of diagnosis, treatment and unintended exposure to healthcare workers. We present a novel extra-pulmonary manifestation of COVID-19 resulting exclusively in COVID-19-induced colitis.

## Case presentation

**Figure 1:**
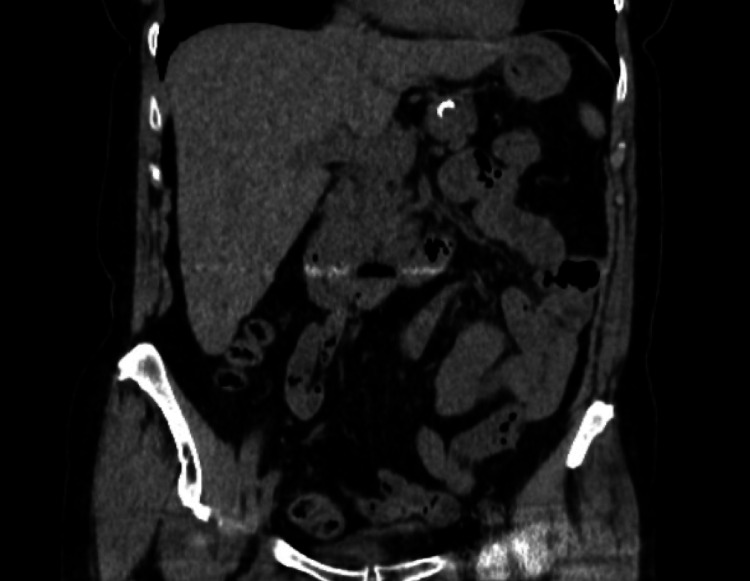
CT abdomen and pelvis showing thickening of the descending colon along with peri-colic fat stranding consistent with acute colitis

An 80-year-old Caucasian woman with a history of hypertension, hyperlipidemia, hypothyroidism, and tobacco use presented to the hospital with a five-day history of low-grade fever, lower abdominal discomfort, nausea, watery diarrhea, and hematochezia. The patient denied any symptoms of anosmia, nocturnal diaphoresis, shortness of breath, cough or chest pain. The patient denied any recent travel or ill contacts, including those with known exposure to COVID-19. Initial laboratory workup was significant for normocytic anemia with a hemoglobin of 11.2 g/dl (baseline hemoglobin of 13 g/dl). Physical examination was significant for a body temperature of 100.6 °F, a blood pressure of 130/65 mmHg, pulse of 83 bpm, respiratory rate of 15 breaths per minute, and oxygen saturation of 96% while breathing ambient air. Lung auscultation was clear bilaterally. Abdominal examination revealed a soft, nondistended abdomen, with positive bowel sounds and mild to moderate tenderness in the lower abdominal quadrants bilaterally. Digital rectal exam revealed bright red blood in the rectal vault. Chest radiography showed no abnormalities. A computed tomography (CT) scan of the abdomen and pelvis on admission revealed thickening of the wall of the descending colon with infiltration of the pericolonic fat consistent with acute colitis (Figure 1). Further workup revealed new-onset lymphopenia of 3x10^3^/mcL. Complete metabolic panel along with liver function tests, lactic acid, lactic acid dehydrogenase (LDH), and coagulation profile were all within normal limits. A complete workup for lymphopenia was negative, including negative HIV testing. C. difficile testing along with rapid nucleic acid amplification testing (NAAT) for influenza A and B were negative. COVID-19 RT-PCR testing returned positive on two occasions on the second and fourth day of hospitalization respectively.

On the second day of admission, rectal bleeding and diarrhea resolved and hemoglobin remained stable without the need for blood transfusion. Mild abdominal discomfort remained throughout the hospitalization. Intravenous fluid hydration and empiric antibiotic therapy were continued through the third day of hospitalization, followed by ceftriaxone and metronidazole until discharge on the fifth day of hospitalization. In the setting of active colitis and symptomatic improvement, colonoscopy was deferred to the outpatient setting. The patient was discharged home with 10 days of metronidazole therapy, in addition to recommendations for the patient to observe 14 days of home self-isolation. At the time of writing, the patient continues to be followed closely without further complication. 

## Discussion

Many aspects of this developing pandemic are not yet fully understood, including transmissibility of the virus, variance in presentation upon hospital admission and the pathophysiology behind various organ system involvement. Per current literature review, our report illustrates the second reported case of COVID-19-induced colitis. Our patient presented with abdominal pain, watery diarrhea and hematochezia consistent with an acute hemorrhagic colitis, without known risk factors or respiratory involvement associated with COVID-19 infection. 

Recently published literature suggests that SARS-CoV-2 has the capability to directly or indirectly damage the digestive system through a viral inflammatory response [4-6]. The proposed mechanism for this is the accumulation of inflammatory cytokines such as IL-6, IL-7, TNF and inflammatory chemokines, causing an overwhelming viremic response with resultant injury to the digestive mucosa [4-7]. Gastrointestinal involvement likely begins at the biochemical level involving ACE2 protein receptors, where it has been shown SARS-CoV-2 interacts with these receptors described by Fei Xiao et al [6]. Immunofluorescent data reveals that the ACE2 protein is abundantly expressed in the glandular cells of gastric, duodenal, colonic and rectal epithelia, which supports entry of SARS-CoV-2 into host cells [6]. SARS-CoV-2 virus can therefore be classified as an enteropathic infection, through its ability to directly affect the intestinal mucosa, thereby causing as in our case, hemorrhagic colitis. Most interestingly, some studies have shown that viral nucleic acid may be detected in stool samples in up to 53.4% of patients [4-6]. These data suggest the possibility of fecal-oral transmission and the importance of increased awareness and precautions required to mitigate viral transmission [8]. 

To our knowledge, this is an uncommon occurrence of SARS-CoV-2 causing direct gastrointestinal involvement resulting in hemorrhagic colitis with other etiologies being virtually excluded. 

## Conclusions

This case report highlights the significance of limiting fecal-oral transmission along with adding to the body of evidence that SARS-CoV-2 has the potential to directly infect the gastrointestinal tract in the absence of pulmonary involvement. With mounting evidence, we underline the importance of instituting appropriate precautions in patients who present with respiratory, digestive or other rare clinical presentations. Healthcare workers must remain vigilant during these trying times in order to protect themselves and patients alike. We encourage further research on the effects of SARS-CoV-2 on the gastrointestinal tract and support the development of improved fecal testing.

## References

[1] Abd El-Aziz TM, Stockand JD (2020). Recent progress and challenges in drug development against COVID-19 coronavirus (SARS-CoV-2) - an update on the status. Infect Genet Evol.

[2] Mungroo MR, Khan NA, Siddiqui R (2020). Novel coronavirus: current understanding of clinical features, diagnosis, pathogenesis, and treatment options. Pathogens.

[3] Pan L, Mu M, Yang P (2020). Clinical characteristics of COVID-19 patients with digestive symptoms in Hubei, China: a descriptive, cross-sectional, multicenter study. Am J Gastroenterol.

[4] Tang A, Tong ZD, Wang HL (2020). Detection of novel coronavirus by RT-PCR in stool specimen from asymptomatic child, China. Emerg Infect Dis.

[5] Xie C, Jiang L, Huang G (2020). Comparison of different samples for 2019 novel coronavirus detection by nucleic acid amplification tests. Int J Infect Dis.

[6] Xiao F, Tang M, Zheng X, Liu Y, Li X, Shan H (2020). Evidence for gastrointestinal infection of SARS-CoV-2. Gastroenterology.

[7] Merad M, Martin JC (2020). Pathological inflammation in patients with COVID-19: a key role for monocytes and macrophages. Nat Rev Immunol.

[8] Holshue ML, DeBolt C, Lindquist S (2020). First case of 2019 novel coronavirus in the United States. N Engl J Med.

